# A radiographic and anthropometric study of the effect of a contoured sandal and foot orthosis on supporting the medial longitudinal arch

**DOI:** 10.1186/s13047-014-0038-5

**Published:** 2014-10-04

**Authors:** Carles Escalona-Marfil, Thomas G McPoil, Rebecca Mellor, Bill Vicenzino

**Affiliations:** Universitat Internacional de Catalunya, Facultad de Medicina y Ciencias de La Salud, Barcelona, Spain; School of Physical Therapy, Ruekert-Hartman College for Health Professions, Regis University, Denver, CO USA; The University of Queensland, School of Health and Rehabilitation Sciences: Physiotherapy, Brisbane, Queensland Australia

**Keywords:** Foot, Orthotic Devices, Shoes, Radiology

## Abstract

**Background:**

In-shoe foot orthoses improve conditions such as plantar heel pain (fasciitis), probably due to their ability to raise the medial longitudinal arch of the foot and lower the stress on the plantar tissues. Increasingly the arch-profile form of the in-shoe foot orthosis is being incorporated into sandal footwear, providing an alternative footwear option for those who require an orthosis. The purpose of this study was to evaluate if a sandal that incorporates the arch-profile of an in-shoe foot orthosis does indeed raise the medial longitudinal arch.

**Methods:**

Three commercially available non-medical devices (contoured and flat sandal, prefabricated in-shoe orthosis) worn by healthy individuals were studied in two independent experiments, one using radiographic measurements in Australia (n = 11, 6 female, age 26.1 ± 4.3 yrs, BMI 22.0 ± 2.4 kg/m^2^) and the other utilising anthropometric measures in the USA (n = 10, 6 female, age 26.3 ± 3.8 yrs, BMI 23.5 ± 3.7 kg/m^2^). A barefoot condition was also measured. Dorsal arch height was measured in both experiments, as well as in subtalar neutral in the anthropometric experiment. One way repeated measures ANOVA with follow up Bonferroni-corrected pairwise comparisons were used to test differences between the conditions (contoured and flat sandal, orthosis, barefoot). Mean difference and 95% confidence intervals (CI) and standardised mean differences (SMD) were also calculated.

**Results:**

The contoured sandal significantly increased dorsal arch height compared to barefoot and flat sandal in both the anthropometric and radiographic experiments with SMD ranging from 0.95 (mean difference 5.1 mm (CI: 0.3, 1.6)) to 1.8 (4.3 mm (1.9, 6.6)). There were small differences between the contoured sandal and orthosis of 1.9 mm (0.6, 3.3) in the radiographic experiment and 1.2 mm (−0.4, 0.9) in the anthropometric experiment. The contoured sandal approximated the subtalar neutral position (0.4 mm (−0.5, 0.7)).

**Conclusions:**

Medial longitudinal arch height is elevated by contoured sandals and approximates subtalar joint neutral position of the foot and that achieved by an orthosis. Practitioners wanting to increase the medial longitudinal arch can do so with either an orthosis or a contoured sandal that includes the raised arch profile form of an orthosis.

## Background

Evidence from systematic reviews is emerging in support of the role of in-shoe foot orthoses for the management of such foot conditions as chronic plantar heel pain, often termed plantar fasciitis [1-3]. These reviews have demonstrated that pre-fabricated or custom foot orthoses provide short-term reduction in pain and improved function in individuals with chronic plantar heel pain. In addition, no difference has been demonstrated in the amount of pain reduction or improved function provided by custom versus pre-fabricated foot orthosis [2,4,5]. This is an important factor that influences the benefit-to-cost ratio for the individual with chronic plantar heel pain if foot orthoses are part of the plan of care.

One reason for the success of foot orthoses in this patient population could be attributed to the fact that individuals with chronic plantar heel pain are more likely to have a more pronated foot posture [6]. The mechanism by which the role of foot orthoses is mediated is thought to occur through altering the orientation of foot bones into a more ideal mechanical alignment, which arguably would optimise loading of the soft tissues of the foot. Previous cadaveric studies have substantiated this proposed mechanism by demonstrating that foot orthoses designed to provide support to the medial longitudinal arch of the midfoot not only improve arch stability [7] but also decrease the strain on the plantar aponeurosis [8].

While the use of a foot orthosis to provide support to the medial longitudinal arch of the midfoot would appear to be justified in the early stages of management for the individual with chronic plantar heel pain, the effectiveness of these devices is regarded to be dependent on compliance with wearing closed in footwear that is able to contain the orthosis. Recently, the foot bed design of the in-shoe foot orthosis, which usually incorporates an arch support, has been used in the development of slip-on sandal footwear to provide support to the midfoot. These slip-on, contoured sandals with a built-in arch support are conceivably more likely to be worn by individuals with chronic plantar heel pain who reside in hot climates in which the use of closed footwear with orthotics is uncomfortable. In addition, slip-on contoured sandals are easier to put on in order to alleviate first step pain on initial weight bearing when first arising in the morning, which is one of the most common symptoms associated with chronic plantar heel pain.

When considering the use of contoured sandals versus foot orthoses in the management program, a key question for the health care provider is whether the support provided by the sandal is equivalent to that provided by a foot orthosis. After a review of the available literature, no studies could be found that have assessed the amount of midfoot support provided by sandal devices that incorporate a built-in arch support in comparison to a foot orthosis. Thus, this study aimed to evaluate through two independent experiments the amount of midfoot support provided by a contoured sandal with built-in arch support in comparison to a pre-fabricated foot orthosis and a flat sandal. Foot posture in barefoot standing was also assessed in both experiments to provide a baseline comparator. One experiment used radiographic measurements of the foot (Australia) whereas the second experiment used non-invasive anthropometric foot measurements (USA) previously described in the literature [9]. Reporting the findings from both experiments in one paper should serve to underpin the veracity of the implications of the findings. In addition to barefoot standing, subtalar joint neutral position was used as a second baseline comparator for the anthropometric foot measurement experiment.

We developed three hypotheses for this investigation. Firstly, we hypothesised that there would be no differences in the amount of midfoot support, as determined by dorsal arch height and navicular height, provided by both the contoured sandal and foot orthosis. Secondly, we hypothesised that both the contoured sandal and foot orthosis would provide a significantly greater degree of midfoot support, as determined by dorsal arch height and navicular height, in comparison to the flat sandal and standing barefoot. Finally, we hypothesised that the amount of midfoot support provided by both the contoured sandal and pre-fabricated foot orthosis, as determined by dorsal arch height, would not be the same when compared to the change in dorsal arch height when measured in subtalar joint neutral position.

## Methods

### Experimental conditions for both experiments

**Figure 1:**
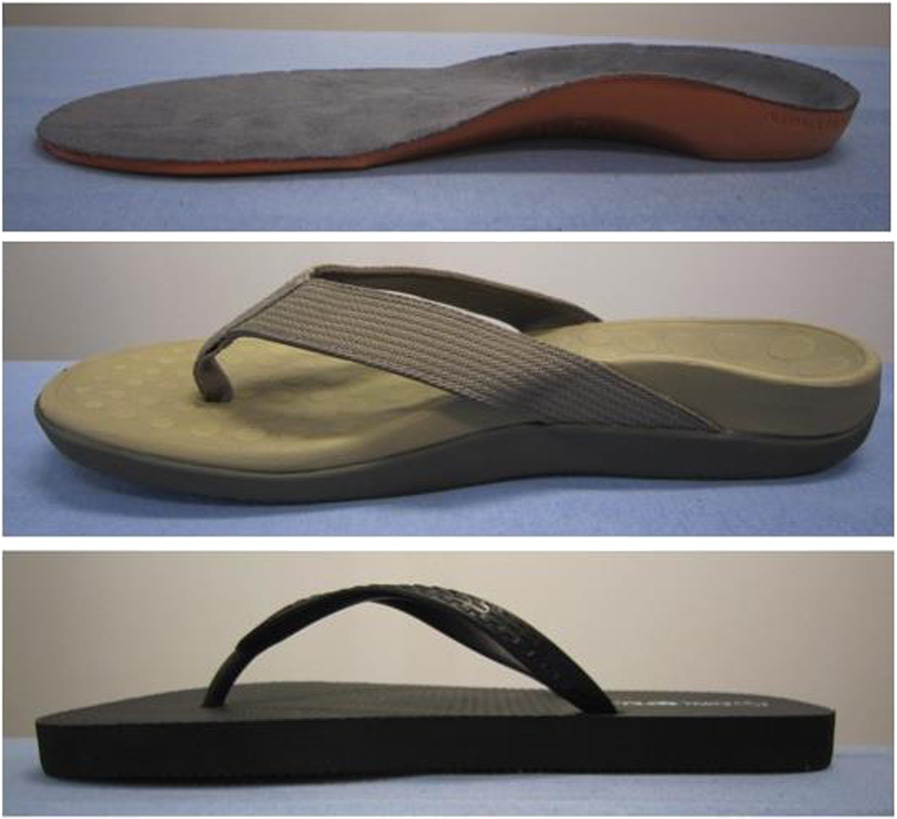
**Devices being studied: contoured sandal, orthosis and flat sandal.**

An unshod condition and three over-the-counter (publicly available without medical prescription) footwear devices were compared (Figure 1). Two of the devices had contoured medial arch support built within the device, whereas the other one was a standard flat sandal (also commonly referred to as a flip flop or thong). The contoured devices were an in-shoe orthosis (Orthaheel, Vionics International, California, USA) and a contoured sandal, which incorporated a similar arch support design (foot bed technology) as the orthosis, but in a slip-on sandal (Orthaheel, Vionics International, California, USA). The manufacturer supplied the devices. The devices were fit to the participants on the basis of comfort and size. The hardness of the three devices assessed in the midfoot region of the device using a Shore A durometer (Rex Gauge, Buffalo Grove, Illinois, USA) were: flat sandal 38, contoured sandal 62, and the orthosis 56.

### Participant selection criteria for both experiments

All participants met the following selection criteria: (i) no history of congenital deformity in the lower extremity or foot; (ii) no previous history of lower extremity or foot fractures; (iii) no systemic diseases that could affect lower extremity or foot posture; (iv) no visible signs of foot pathology in either foot, including non-reducible claw or hammer toes, hallux valgus, hallux limitus, or hallux rigidus; and (v) no history of trauma or pain to either foot, lower extremity, or lumbosacral region at least 6 months prior to the start of the investigation.

### Radiographic experiment methods

Participants for the radiographic experiment were recruited from the University of Queensland staff and student body in Brisbane, Australia. All participants gave written informed consent before participating in the study, and approval for the study was obtained from the University of Queensland Human Research Ethics Committee (#201200068).

**Figure 2:**
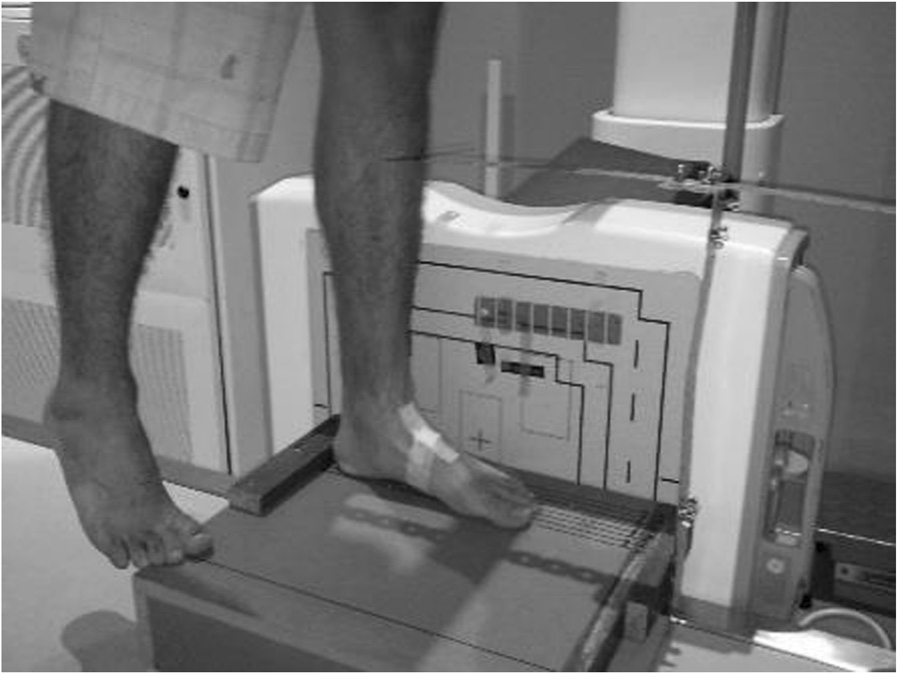
**Platform constructed in order to standardize position of foot and leg relative to the X-ray camera and plate.**

All radiographs were taken on the same x-ray machine (GE Definium 6000, Siemens, AL01C) in one centre. Scanning parameters were: 60 kV/2mAs. A custom-made wooden platform was constructed in order to consistently place the foot within the imaging field, thereby reducing error from such sources as repositioning of the foot and from parallax error (Figure 2). The platform measured 40 cm × 35 cm × 10 cm. Attached to the rear and superior aspect of the platform was a 4.2 cm wide block, which was used to situate the posterior aspect of the heel. Orthogonal to the block, a reference grid of eight parallel lines was marked onto the upper surface of the platform. Along the centre line, four fine wire nails were imbedded at known distances from the rear block (12 cm, 17.5 cm, 24 cm and 30 cm). The position of the tibial tuberosity in the sagittal plane relative to the platform was standardised by means of a vertically extended bar. Placed within easy reach of the participant was a stable horizontal bar that was used to maintain balance (by fingertip touch), but not reduce weight bearing during the single limb weight bearing position.

Four weight-bearing lateral radiographs were taken of the left foot in: (i) an unshod barefoot condition, (ii) while wearing a contoured sandal (Orthaheel, Vionics International, California, USA), (iii) non-contoured flat sandal, as well as (iv) standing on a full length orthosis (Orthaheel, Vionics International, California, USA) not inserted into a shoe.

A standardised protocol was adopted in order to reduce experimental error in measuring the radiographic images. The posterior mid-point of the calcaneus was located and marked with a pen, 1 cm from the floor. The total foot length was measured and divided in half to locate and mark the dorsal arch height of the mid-foot, at which a radio-opaque marker was taped, thereby providing the reference point used to measure the Dorsal Arch Height at the mid-foot on the radiograph. The plantar midline of the foot was identified and marked (by standing on a length of raised metal to produce a transient indentation in the surface of the skin) with two radio-opaque markers, one at the level of the mid-heel and the other at the level of the second metatarsal head.

Participants were then positioned in standing with the left foot fully weight bearing on the wooden platform. To help maintain balance, the right toe lightly touched the platform outside the imaging field posterior to the left foot. The lateral edge of the left foot was aligned to the x-ray plate, and the midline of the calcaneus and the space between the second and third toes were aligned along one of the platform grid lines (appropriate to foot size). Participants were asked to gently touch the vertically extended upright bar with their tibial tuberosity, so as to have the same tibial inclination in the sagittal plane for all four conditions. This protocol aimed to standardise foot placement on the repeated radiographs of the different interventions.

### Anthropometric experiment methods

Participants for the anthropometric experiment were recruited from the Regis University staff and student body in Denver, Colorado, USA. Since previous research has reported that individuals with chronic plantar heel pain are more likely to have a more pronated foot type [6] only those volunteers that had a change in midfoot width of greater than 12 mm change in the width of the midfoot from non-weight bearing to weight bearing, measured at 50% of the total foot length, were asked to participate. The procedure used to measure midfoot width has been previously described by McPoil et al. [9]. Previous research has shown that an increase in midfoot width between non-weight bearing and weight bearing is associated with foot posture [10]. All participants gave written informed consent before participating in the study, and approval for the study was obtained from the Regis University Institutional Review Board for the Ethical Treatment of Human Subjects (#11-246). Prior to the anthropometric measurements, each participant’s age, height, and body weight were recorded.

**Figure 3:**
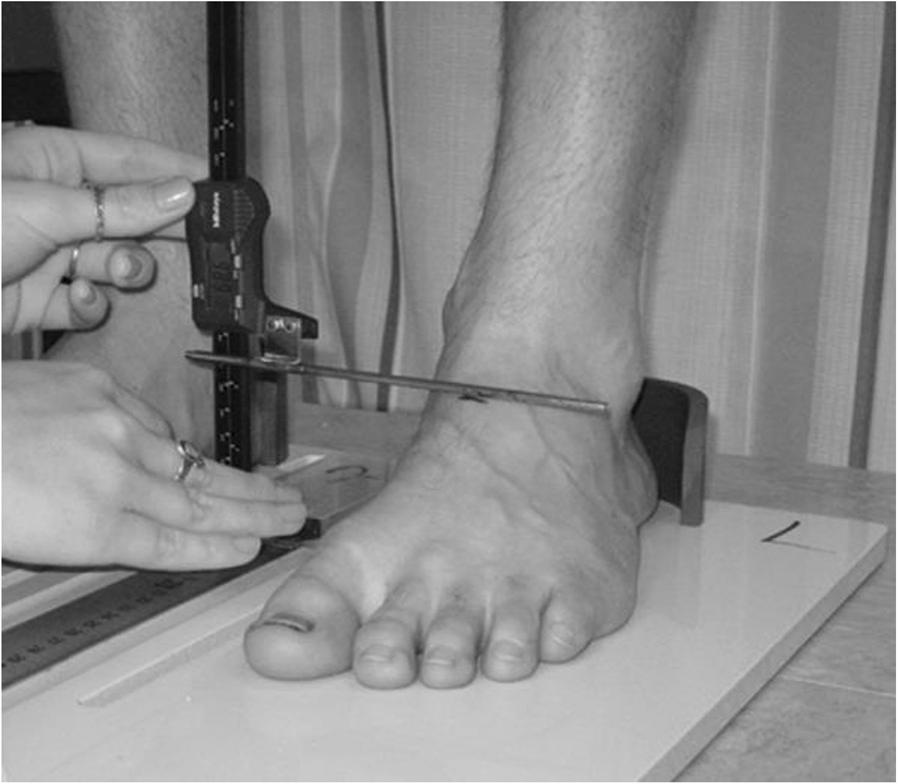
**Measurement device for dorsal arch height using a modified digital caliper (Model #700-126, Mitutoyo America Corp, Aurora, IL 60502).** A 1.2 × 5.0 × 10.0 cm plastic block holds the caliper vertically. The arch height is measured by a sliding metal rod extension of the caliper arm.

Mid foot or dorsal arch height was measured with a weight bearing arch height gauge, which consisted of a digital caliper (Model #700-126, Mitutoyo America Corp, Aurora, IL 60502) with the fixed point attached to a 12 × 50 × 100 mm plastic block to hold the caliper in a vertical position and a sliding metal rod attached to the moving point of the caliper to permit the assessment of dorsal arch height (see Figure 3) [9].

**Figure 4:**
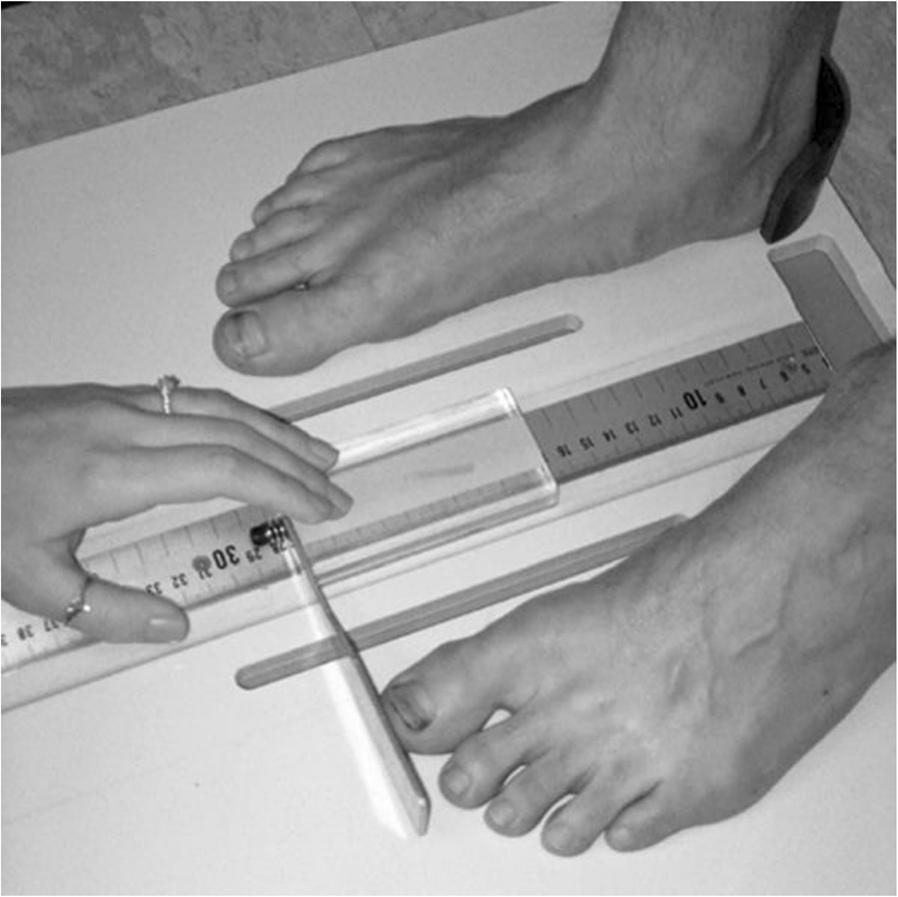
**Platform used to standardize the placement of the devices and feet as well as to measure foot length by means of the ruler and a sliding bar.**

Each participant was asked to stand on an elevated table that was positioned approximately 61 cm from the floor and to place their feet onto a specially constructed Foot Assessment Platform previously described (Figure 4) [9]. The participant was positioned on the platform with both heels placed in left and right heel cups that were 15.24 cm apart. Next, the medial prominences of the first metatarsal heads of both feet were positioned so they were just touching a plastic bar to ensure consistent forefoot placement on the platform. Once the participant was properly positioned on the platform, the participant was instructed to relax and place equal weight on both feet so that the weight bearing measurements could be obtained. Total foot length was first measured by placing the sliding bar on the centered metal ruler attached to the platform and moving the bar to just touch the longest toe, usually the hallux, of each foot (see Figure 4). Next, the dorsal arch height at 50% of total foot length was measured bilaterally using the weight bearing arch height gauge previously described. To determine 50% of total foot length, the previously measured total foot length was divided in half and the dorsum of both feet were marked at the 50% length point using a water-soluble pen. The sliding metal rod of the weight bearing height gauge was then positioned over the 50% length for both feet (see Figure 3) and the dorsal arch height measured. Each participant’s foot was then placed in subtalar joint neutral by asking the participant to elevate and lower the medial longitudinal arch of one foot followed by the other foot while the investigator palpated the medial and lateral aspect of the head of the talus in relation to the navicular bone. When the investigator felt congruency between the head of the talus and the navicular bone (subtalar joint neutral position) in both feet, the participant was instructed to maintain that position while the dorsal arch height was measured for both feet.

Next, each participant was asked to step off the platform so that the flat sandal could be positioned on the platform. The participant was then asked to stand on the flat sandal so their feet could be placed in the same position on the Foot Assessment Board as previously described. Once positioned, the dorsal arch height measurement at the 50% length mark was repeated on both feet with the subject standing with their feet relaxed on the flat sandal. The same procedure was then repeated for the contoured sandal and orthosis conditions with the order of testing for the three conditions, flat sandal, contoured sandal, and orthosis, randomised.

Navicular height was not assessed in the anthropometric experiment. While an attempt was made to locate and mark the navicular tuberosity while the participant was standing barefoot, when the participant stood on both the contoured sandal and orthosis conditions the curve of the arch piece in both devices created a distortion of the soft tissue in the medial longitudinal arch region that affected the position of the skin marking.

### Data management and analysis

**Figure 5:**
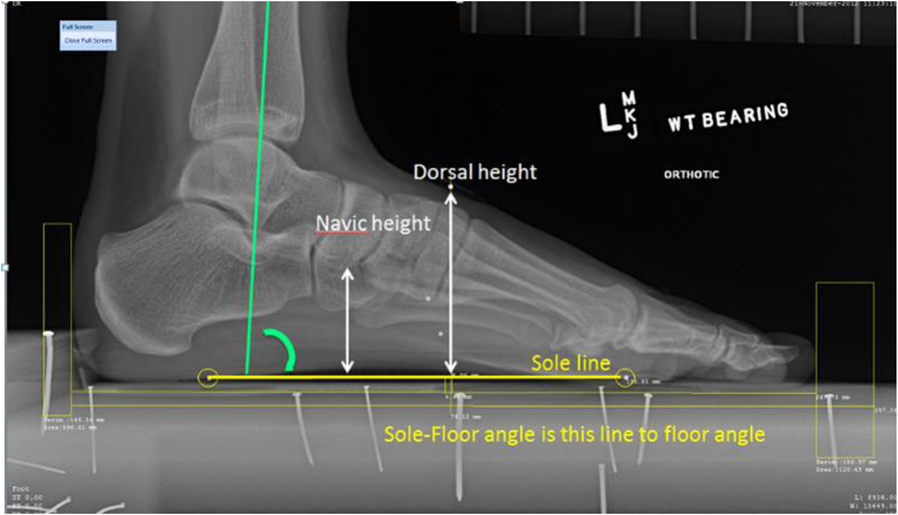
**The reference sole line against which are measured the two linear measures approximating mid foot height and angular measures of the tibia on the foot.** The angle of the foot to the platform was measured between the sole line and the nail heads within the platform.

For the radiographic experiment, the lateral radiographs were analysed by a single researcher (CE), and viewed using the MicroDicom Viewing software program (Version 0.7.6, http://www.microdicom.com/, Sofia, Bulgaria). Two linear measurements of mid foot height and two angular measurements of the position of the foot and tibia in the sagittal plane were made (Figure 5). The two radio-opaque markers under the heel and second metatarsal head were located and a line drawn between them, identified as the sole line against which the linear and angular measures were referenced. The dorsal height was measured perpendicularly from the sole line and the dorsal radio-opaque marker located at the mid-foot point. The navicular height was likewise measured perpendicularly between the sole line and the most inferior aspect of the navicular bone. The linear measures were calibrated against the known distances between the nail heads located within the platform.

The angle of the sole line relative to the nail heads located in the platform was also measured as it represents the position of the plantar surface of the foot to the floor in the sagittal plane position (e.g. relative plantarflexion/dorsiflexion of foot to floor). The tibia-sole angle was the angle between the sole line and a line representing the longitudinal axis of the tibia, which is an index of tibia-foot dorsiflexion-plantarflexion.

The inter-rater reliability of measuring these parameters from the radiographs was evaluated by having another investigator also perform the measurements. The level of reliability was then calculated with Intraclass Correlation Coefficients (ICC_1,2_) and their 95% confidence intervals, the Standard Error of the Measurement and the Minimal Detectable Change at 95% confidence. The latter two are in the unit of the measurements and provide an estimate of the amount of error between raters. The recommendations of Landis and Koch [11] were used to rate the degree of reliability with <0.2 being slight, 0.21 to 0.4 as fair, 0.41 to 0.60 as moderate, 0.61 to 0.8 as substantial and >0.8 as almost perfect.

An estimate of the error in the radiographic measurements was also calculated with a Limits of Agreement approach [12] from the data on foot length collected both from a caliper measure and the radiograph in the participants in the x-ray experiment. The amount of error (difference between caliper and radiograph foot length in the radiographic experiment cohort) and its confidence intervals and Limits of Agreement (LoA) confidence was calculated and used to provide an outside estimate of the measurement error in order to context any differences between devices.

Individual data was visually inspected and presented in a table as the mean (standard deviation) for all conditions (barefoot, flat sandal, contoured sandal, and orthosis). Differences between the conditions were evaluated with a repeated measure ANOVA (*p*-level of 0.05). Significant main effects were followed up with Bonferroni-corrected pairwise comparisons. Mean differences (95% confidence intervals (CI)) and *p*-value for all pairwise comparisons were tabulated. Standardized mean difference (SMD) representing the effect size between pairs of conditions was calculated from the mean difference and their pooled standard deviation. Interpretation of SMD was based on Hopkins’ [13] classification of trivial (<0.2), small (0.2–0.6), medium (0.61–1.2) and large (>1.2).

For the anthropometric experiment, the dorsal height measurements recorded with each participant standing on the flat sandal, contoured sandal, and orthosis were adjusted to account for the added height of each device using radiographic data (average height for each of the three devices based on radiographic data).

Data were analysed and presented in the same way as the radiographic data in that the mean (standard deviation) for all conditions (barefoot in relaxed stance, barefoot with the subtalar joint in neutral position, flat sandal, contoured sandal, foot orthosis). Differences between the conditions were evaluated with a repeated measures ANOVA and Bonferroni-corrected pairwise comparisons in following up significant main effects. Standardised mean difference (SMD) representing the effect size between pairs of conditions was calculated from the mean difference and their pooled standard deviation. Interpretation of SMD was based on Hopkins’ (15) classification of trivial (<0.2), small (0.2–0.6), medium (0.61–1.2) and large (>1.2).

## Results

### Radiographic experiment

Eleven participants (6 female), with a mean age of 26.1 ± 4.3 yrs and BMI of 22.0 ± 2.4 kg/m^2^ consented to participate in the radiographic experiment (Table 1).

**Table 1:** **Participant details for the x-ray experiment**

	**Female (n = 6)**	**Male (n = 5)**	**Total (n = 11)**
Age, years	24.8 ± 2.6 (22–28)	27.6 ± 5.7 (22–35)	26.1 ± 4.3 (22–35)
Weight, kg	60.0 ± 5.9 (54–70)	69.4 ± 12.5 (53–84)	64.3 ± 10.2 (53–84)
Height, cm	165.7 ± 5.9 (155–170)	175.8 ± 6.3 (166–183)	170.3 ± 7.8 (155–183)
BMI, kg/m^2^	21.9 ± 1.7 (19.8-24.2)	22.3 ± 3 (19.2-25.5)	22.1 ± 2.3 (19.2-25.5)

Values are mean ± SD (range).

The linear measures of dorsal and navicular height and all angular measures were almost perfectly reliable with low levels of error (Table 2).

**Table 2:** **Reliability indices (intra-class coefficients (95% confidence interval), standard error of measurement) and minimal detectable change at 95% confidence (linear measures in mm and angle/pitch in degree) for x-ray measurements**

	**Inter-rater**	**(95% confidence interval)**	**SEM**	**MDC95**
Dorsal height	0.997	(0.993 to 0.999)	0.217	0.6
Navicular height	0.994	(0.986 to 0.998)	0.394	1.1
Tibia-sole angle	0.985	(0.961 to 0.994)	0.282	0.8
Sole angle	0.906	(0.763 to 0.963)	0.327	0.9
Foot length	0.986	(0.966 to 0.995)	7.637	21.2

The difference between caliper and radiographic measurement of foot length for the cohort was 2.5 mm (95% CI: 1.1 to 3.9; LoA: −6.34 to 1.34). This represents approximately 1% of error for an average foot length of 255 mm (95% CI: 248 to 262), which is likely an overestimate of any error in the x-ray data when considering that the linear measurements of arch height or navicular height are in the order 66 mm and 39 mm respectively.

**Figure 6:**
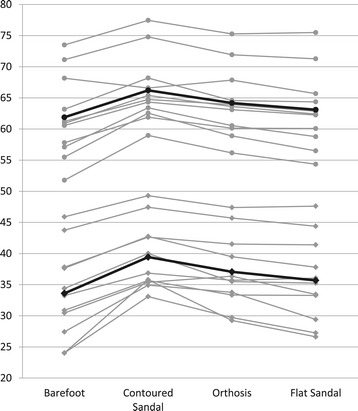
**Individual patient data (mm) for the dorsal arch height (circle) and navicular height (square) with darker markers and lines indicating condition mean.**

Individual patient data for the medial longitudinal arch measures are shown in Figure 6, while Table 3 includes the mean (standard deviation) descriptives for all conditions and Table 4 presents the point estimates of effect for all pairwise comparisons.

**Table 3:** **Condition mean (SD) data; linear in mm and angular in degrees for the x-ray experiment**

	**Barefoot**	**Flat sandal**	**Contoured sandal**	**Orthosis**
Arch height	61.9 (6.8)	63.0 (6.3)	66.2 (5.4)	64.3 (5.6)
Navicular height	33.6 (7.4)	35.6 (6.8)	39.5 (5.3)	37.1 (5.9)
Sole to floor angle*	0.1 (0.3)	−1.6 (0.5)	0.1 (0.3)	1.5 (0.7)
Tibia to sole angle	84.0 (3.2)	83.6 (4.9)	86.0 (3.9)	84.3 (4.0)
Foot length	253.7 (12.4)	254.3 (13.1)	253.0 (12.7)	253.5 (11.8)

*-ve value indicates dorsiflexion.

**Table 4:** **Pairwise comparisons expressed as mean difference and 95% confidence intervals (CI),** ***p*** **-value and standardised mean difference (SMD) for linear measures in mm and angular measures in degrees**

		**Contoured sandal**	**Contoured sandal**	**Contoured sandal**	**Orthosis**	**Orthosis**	**Flat sandal**
		**Barefoot**	**Flat sandal**	**Orthosis**	**Barefoot**	**Flat sandal**	**Barefoot**
Dorsal height	MD	4.3	3.2	1.9	2.4	1.3	1.1
	95% CI	(1.9 to 6.6)	(1.4 to 5.0)	(0.6 to 3.3)	(1.0 to 3.7)	(0.2 to 2.4)	(0.1 to 2.3)
	*P*-value	0.001	0.001	0.006	0.001	0.020	0.085
	SMD	1.8	1.7	1.4	1.7	1.2	0.9
Navicular height	MD	5.9	3.9	2.4	3.6	1.6	2.0
	95% CI	(3.2 to 8.6)	(1.4 to 6.4)	(0.2 to 4.5)	(1.4 to 5.7)	(−0.1 to 3.2)	(0.8 to 3.2)
	*P*-value	<0.001	0.003	0.028	0.002	0.077	0.001
	SMD	2.2	1.6	1.1	1.6	0.9	1.7
Sole to floor angle	MD	1.4	3.0	1.4	0.0	1.6	1.6
	95% CI	(0.6 to 2.2)	(2.2 to 3.8)	(0.6 to 2.2)	(−0.4 to 0.4)	(1.0 to 2.3)	(1.1 to 2.1)
	*P*-value	0.001	<0.001 3.9	0.001	1.000	<0.001 2.4	<0.001 3.2
	SMD	1.7		1.7	0		
Tibia to sole angle	MD	2.0	2.4	1.7	0.3	0.6	0.4
	95% CI	(0.3 to 3.7)	(0.2 to 4.5)	(0.6 to 2.9)	(−1.5 to 0.9)	(−2.7 to 1.4)	(−2.2 to 2.9)
	*P*-value	0.020	0.031	0.004	1.000	1.000	1.000
	SMD	1.2	1.1	1.5	0.2	0.3	0.1
Foot length	MD	0.7	1.3	0.5	0.2	−0.8	−0.6
	95% CI	(−1.3 to 2.7)	(−0.4 to 3.0)	(−1.7 to 2.7)	(−2.1 to 2.5)	(−3.1 to 1.5)	(−2.1 to 0.9)
	*P*-value	1.000	0.200	1.000	1.000	1.000	1.000
	SMD	0.4	0.8	0.2	0.1	−0.4	−0.4

Of the linear measures taken from radiographs there was a significant main effect for the dorsal mid foot height (F_3,30_ = 28.8, *p* < 0.001) and the navicular height (F_3,30_ = 30.3, *p* < 0.001), but not for the foot length (F_3,27_ = 1.56, *p* = 0.222). The greatest differences in mid foot height as measured from the sole line, were between the contoured sandal and barefoot condition (mean differences (SMD): dorsal height 4.3 mm (1.8), navicular height 5.9 mm (2.2)). The increases in dorsal and navicular heights are approximately 7% and 18% of the barefoot height, respectively. The contoured sandal produced greater differences in dorsal and navicular height than did the orthosis (SMD: 1.4 and 1.1 respectively). The orthosis, in turn, had greater increases in dorsal and navicular heights than the flat sandal or barefoot conditions (SMD range: 1.2 to 1.7) with the exception of the navicular height in comparison to the flat sandal (SMD: 0.9, *p* = 0.077). Interestingly, the flat sandal resulted in a significantly higher navicular bone than in barefoot (mean difference: 2.0 mm, SMD: 1.7, *p* = 0.001).

The Sole Line was used as the basis for the dorsal and navicular height measures as well as for the foot-floor and tibia-foot angle. The angle of the sole to the floor was different between conditions (F_3,30_ = 70.55, *p* < 0.001), with the largest difference between the contoured sandal and flat sandal (mean difference: 3.0°, SMD: 3.9, *p* < 0.001), which is likely a combination of the dorsiflexed position in the flat sandal relative to barefoot (mean difference: 1.6°, SMD: 3.2, *p* < 0.001) and the plantarflexed position in the contoured sandal relative to barefoot (mean difference: 1.4°, SMD: 1.7, *p* = 0.001). Interestingly, the orthosis does not have a different angular profile at the sole line compared to barefoot (mean difference: 0.0, *p* = 1.0, SMD: 0.0).

The contoured sandal produced relative plantar flexion of the tibia on the sole line (foot) when compared to all other conditions (F_3,30_ = 6.78, *p* = 0.001, SMD range: 1.1 to 1.5), which likely reflects our requirement for participants to maintain the tibial tuberosity in the same sagittal plane position between the different conditions and the plantarflexion of the foot on the floor (as seen from the sole to floor angle).

### Anthropometric experiment

Ten participants (6 female), with a mean age of 26.3 ± 3.8 yrs and BMI of 23.5 ± 3.7 kg/m^2^ consented to participate in the anthropometric experiment (Table 5). The mean change in midfoot width was 14.5 ± 2.5 mm.

**Table 5:** **Participant details for the anthropometric study**

	**Female (n = 6)**	**Male (n = 4)**	**Total (n = 10)**
Age, years	27.5 ± 4.3 (23 to 35)	24.5 ± 2.4 (23 to 28)	26.3 ± 3.8 (23 to 35)
Weight, kg	57.3 ± 10.2 (45.4 to 74.8)	74.7 ± 9.0 (61.2 to 79.4)	64.3 ± 12.9 (45.4 to 79.4)
Height, cm	160.8 ± 5.3 (152.4 to 167.6)	171.6 ± 5.5 (167.6 to 179.7)	165.1 ± 7.5 (152.4 to 179.7)
BMI, kg/m^2^	22.1 ± 3.6 (19.5 to 29.2)	25.4 ± 3.3 (21.1 to 28.3)	23.5 ± 3.7 (19.5 to 29.2)

Values are mean ± SD (range).

All anthropometric measurements were obtained by the same investigator (TGM). This same investigator has previously demonstrated high levels of intra-rater and inter-rater reliability with the same measurements used in the current study [14].

The repeated measures ANOVA identified a significant main effect for differences between the conditions (F_4,28_ = 28.4, *p* < 0.001). Data including point estimates of effect are shown in Table 6. The contoured sandal had significantly greater arch height than all other conditions (mean difference range: 0.4 to 7.5 mm, *p* < 0.001, SMD range: 0.1 to 1.6), except subtalar joint neutral positioning (mean difference: 0.3 mm, *p* = 0.996). The orthosis showed a similar pattern with significant differences to barefoot (mean difference: 3.8 mm, *p* = 0.002, SMD: 0.72) and flat sandal (mean difference = 6.3 mm, *p* < 0.001, SMD = 1.34), but not subtalar joint neutral (mean difference: 0.9 mm, *p* = 0.910). The flat sandal was not different to barefoot (mean difference: 2.5 mm, *p* = 0.104), but both the flat sandal and barefoot measures were different to subtalar joint neutral (mean difference: 7.1 and 4.7 mm, *p* < 0.001, SMD: 1.6 and 0.9). Of the three devices assessed, the contoured sandal most closely replicated the posture of the foot when placed in subtalar joint neutral position.

**Table 6:** **Condition mean (SD) data followed by pairwise comparisons expressed as mean difference and 95% confidence intervals (CI),** ***p*** **-value and standardised mean difference (SMD) for the anthropometric study**

**Condition**	**Arch height, mm**	
Barefoot	55.4 (5.4)	
Subtalar joint neutral	59.9 (4.7)	
Flat sandal	52.9 (5.3)	
Contoured sandal	60.2 (5.4)	
Orthosis	59.2 (5.6)	
**Condition**	**Comparator**	**Mean difference (95% CI), p-value, SMD**
Contoured sandal	Barefoot	5.1 (0.3 to 1.6), <0.001, 1.0
Contoured sandal	Flat sandal	7.5 (0.9 to 2.3), <0.001, 1.6
Contoured sandal	Orthosis	1.2 (−0.4 to 0.9), 0.730, 0.2
Contoured sandal	Subtalar joint neutral	0.4 (−0.5 to 0.7), 0.996, 0.9
Orthosis	Barefoot	3.8 (0.8 to 1.4), 0.002, 0.7
Orthosis	Flat sandal	6.3 (0.7 to 2.0), <0.001, 1.3
Orthosis	Subtalar joint neutral	−0.9 (−0.8 to 0.5), 0.910, −0.2
Flat sandal	Barefoot	−2.5 (−1.2 to 0.1), 0.104, −0.5
Flat sandal	Subtalar joint neutral	−7.1 (−2.4 to −30.9), <0.001, −1.64
Barefoot	Subtalar joint neutral	−4.7 (−1.6 to −0.3), <0.001, −0.9

## Discussion

The purpose of this study was to compare the differences in the amount of midfoot support, as determined by the dorsal arch height and/or navicular height, provided by a contoured sandal with built-in arch support in comparison to a pre-fabricated foot orthosis and a flat sandal. To address this purpose two independent experiments were undertaken across two international sites (University of Queensland, Australia and Regis University, Colorado, USA), both analysing two hypotheses. Addressing the first hypothesis, we found that there was no difference in arch and/or navicular height between contoured sandal and orthosis in the anthropometric study, but that the arch and/or navicular height was higher in a contoured sandal in the radiographic study. This finding could be attributed to the contoured sandal being approximately 10% harder than the orthosis, but of similar shape in the arch region. The second hypothesis proposed that the contoured sandal and orthosis would exhibit higher arch and/or navicular height than the flat sandal and barefoot conditions. Both experiments showed that the contoured sandal exhibited greater arch and/or navicular height than the barefoot condition, with arch height being 6.5% and 9% higher in radiographic and anthropometric experiments respectively and navicular height 15% higher in the radiographic experiment. The orthosis was associated with a 4-6% higher arch height in both experiments and a 9% higher navicular height in the radiographic experiment. That similar results were obtained from two experiments with different methods performed independently across two different sites underscored the veracity of the findings.

A third hypothesis posited that the contoured sandal and orthosis would position differently the arch/navicular bone when referenced to the subtalar joint neutral position. This hypothesis was not supported by the anthropometric experiment, because it showed that there was no difference in arch/navicular height between contoured sandal, orthosis and subtalar joint neutral position. While previous research has demonstrated that the rearfoot rarely functions about subtalar neutral position during stance phase while walking [15], subtalar joint neutral position provides a clinical reference as to whether the rearfoot is pronated or supinated. The fact the contoured sandal and orthosis repositioned the dorsal arch height nearer to that of the subtalar joint neutral position, implies that these devices might optimize foot posture, assuming that subtalar joint neutral is the position where joint and soft tissue mechanics are optimal. Notwithstanding this, it is also important to remember that only static posture was studied herein and that inferences to gait should be made with caution.

Both the radiographic and anthropometric measurements showed that the contoured sandal significantly increased arch height compared with the barefoot condition. The radiographs also showed a relative plantarflexion of the foot relative to the floor and of the tibia on the foot. If the contoured sandal were to induce plantarflexion within the foot it would be reasonable to expect the foot length to reduce, which it did not. Thus, we would speculate that the increase in arch height likely occurred through a change in foot orientation in either the transverse or frontal planes rather than the sagittal plane. The orthosis had similar differences as the contoured sandal in arch/navicular height, with the main exception being that it did not change foot on floor or tibia on foot plantarflexion relative to barefoot (and flat sandal). The fact that an orthosis is usually worn in a shoe and that shoes usually have a higher heel relative to forefoot (i.e. plantarflexed foot bed) it is likely that there would be a similar mechanical effect of an in-shoe foot orthosis in-situ to that of the contoured sandal. Nevertheless, an interesting finding is the difference between devices in arch height, which might be interpreted as arch height being a function of talo-crural plantarflexion. That is, there is increasing arch/navicular height relative to the sole of the foot with increasing plantarflexion.

When interpreting the findings from this study it is important to realise that we did not measure changes in pain and disability in a symptomatic group. We do not know if the amount of change in arch/navicular height engendered by the contoured sandals is clinically meaningful in changing pain and disability in symptomatic participants. In this preliminary study of the contoured sandals we purposely selected asymptomatic healthy participants in order to remove any possible influence of pain on the x-ray or anthropometric measurement process. Follow up studies to establish if the contoured sandals influence pain, disability and foot posture are now required.

We have also assumed that the measure of arch height is a measure of arch/midfoot support, which is not an unreasonable supposition when considering there is an arch-shaped build up on the medial side of the devices or footwear at approximately their mid-point (and corresponding to the midfoot region). Interestingly, we found that despite the difference in selection criteria for the anthropometric experiment in which those with a greater mid foot width mobility were only included, there was a remarkable similarity in findings on arch height. This infers that the influence of the device on the arch might well be independent of the foot’s characteristics (e.g. pronated, mobile or not) and provides a basis for further research. It might also implicate other mechanisms by which the device could influence the shape/posture of the foot. For example, contoured foot orthoses have been shown to reduce plantar heel pressures [16,17] and increased plantar pressures have been implicated in chronic plantar heel pain [18].

A strength of this study is that we carefully constrained the set-up of the foot within the imaging field so as to minimise sources of error between the repeated measures for testing the devices and barefoot conditions. We also conducted an inter-rater reliability assessment of radiographic measurements and found acceptable reliability. As would be expected, we also showed that there is an element of measurement error in the measurements derived from the radiographs. A feature of our study is that we estimated the error between the caliper measure of foot length and the x-ray measure of foot length and showed that it was reasonably small. The fact that the anthropometric experiment found similar differences between the barefoot and both contoured devices (sandal and orthosis) is an indication that there is a real effect despite some measurement error on the x-ray experiment. In addition, the finding that the differences between the contoured devices and the flat sandal on radiograph measures were less than in the anthropometric experiment indicates that the radiographic measures are reasonable estimates of changes induced by the devices.

## Conclusions

In conclusion, the results of this study demonstrate that a contoured sandal, designed with a similar foot bed as a pre-fabricated foot orthosis, can provide the same degree of support to the arch/midfoot region as a pre-fabricated orthosis. These findings provide clinicians with the knowledge that when managing individuals with chronic plantar heel pain who reside in hot climates and prefer not to wear shoes with orthoses because of discomfort, the amount of support to the medial longitudinal arch provided by a contoured sandal is equivalent to the support provided by a pre-fabricated orthosis.
